# Pernicious and Threatening *Plasmodium vivax* as Reality

**DOI:** 10.4269/ajtmh.14-0111

**Published:** 2014-07-02

**Authors:** J. Kevin Baird

**Affiliations:** Eijkman-Oxford Clinical Research Unit, Jakarta, Indonesia; Centre for Tropical Medicine, Nuffield Department of Medicine, University of Oxford, Oxford, United Kingdom

The report by Quispe and others^1^ in this issue of the *American Journal of Tropical Medicine and Hygiene* adds to the substantial and growing body of evidence that a diagnosis of *Plasmodium vivax* malaria often occurs with serious and threatening illness. The retrospective study by Quispe and others^1^ of malaria admissions to a hospital in coastal Peru comes with assurance of no confounding by *P. falciparum* by virtue of its absence from the region. About one-quarter of patients were classified as critically ill with severe anemia, shock, lung injury, renal failure, and cerebral syndromes, and two patients (about 8% of the critically ill) did not survive. Essentially similar rates and findings have come from numerous hospital-based studies from all across the global reach of endemic vivax malaria transmission.^2^ Quispe and others^1^ express appropriate caution regarding undiagnosed comorbidities that may have exacerbated illness in some of those patients.

There is no firm understanding of pathogenesis in vivax malaria. The roles of comorbidities in serious and fatal disease with a diagnosis of *P. vivax* require more thorough investigation than has yet been reported.^3^ The same issue, indeed, haunts mortality associated with a diagnosis of falciparum malaria.^4^ Undiagnosed disease, be it infectious or not, occurs in relative abundance in rural tropical settings and may exacerbate or be exacerbated by acute vivax or falciparum malaria. Real world complexity imposes ambiguities regarding the relationship between threatening illness and the parasite *per se*. Nonetheless, acceptance of the reality of vivax malaria as often provoking pernicious and threatening illness must not await demonstration of the mechanisms or cofactors. The weight of evidence now available leaves no doubt that vivax malaria in many settings often occurs in association with a pernicious and threatening course of illness, which does not assign cause and effect but instead, acknowledges real consequences without regard to their specific genesis.

The false aegis of inherent harmlessness in *P. vivax* resulted in a 60-year hiatus in biological and clinical research as well as public health policy and practice.^2^ We now face a wide range of very serious gaps in understanding, effective research, and clinical tools for dealing with this very serious threat affecting 2.5 billion people.^5^,^6^ The dawning understanding of vivax malaria as pernicious and threatening profoundly impacts the management of malaria as both a clinical and global health problem.

A single entity called the hypnozoite demands a wholly different approach from conventional *P. falciparum*-centric strategic postures in research and practice aimed at malaria. The hypnozoite of *P. vivax* completely transforms the landscape of scientific and strategic thinking in the epidemiology, prevention, control, diagnosis, and treatment of malaria. An inoculation of *P. vivax* sporozoites by a single anopheline bite seeds the liver with a brood of hypnozoites. These hypnozoites remain latent for periods ranging from 3 weeks to 3 years. When they do emerge, they tend to do so in rapid succession at intervals of 3 or 4 weeks. At least three relapses are very common, and up to 20 relapses within 2 years have been documented. Among cohorts in Thailand and Indonesia,^7^–^9^ the incidence density of first relapse in the 2 months after patency was about five attacks per person-year. African malariologists will recognize that rate as approximating that by sporozoite-borne *P. falciparum* in the most heavily malarious regions.

The hypnozoite in the liver may thus be recognized as a very significant reservoir of infection of blood. A patient diagnosed with acute vivax malaria stands at very high risk of suffering subsequent attacks in quick succession with deepening risk of serious illness along with opportunities for transmission to others. Among the many therapies applied with good safety and efficacy against the acute attack, none affect the hypnozoite. A single drug kills hypnozoites: primaquine. This fact—and the ability of primaquine to provoke a potentially lethal acute hemolytic anemia in patients having an inborn deficiency of glucose-6-phosphate dehydrogenase (G6PD)—imposes very serious obstacles to successful management of vivax malaria as a clinical and public health problem.

Quispe and others^1^ allude to one of the most serious problems: pregnant women and their infants. A single attack of vivax malaria in the first trimester of pregnancy elevated the risk of spontaneous abortion, stillbirth, or low birth weight by a factor of four.^10^ Infants were the most vulnerable to onset of severe anemia associated with acute vivax malaria.^11^ Pregnant or lactating women and their infants, among the most vulnerable to serious complications with vivax malaria, cannot receive primaquine therapy. Despite being exposed to holoendemic-like attack rates and high risk of very serious consequences, there has been no work to conceive, evaluate, optimize, and validate chemotherapeutic or chemopreventive strategies for pregnant women and infants diagnosed with *P. vivax* malaria. The absence of evidence-based guidance for this serious problem should be considered an urgent global health problem in need of immediate attention.

Furthermore, most patients diagnosed with G6PD deficiency cannot safely receive primaquine therapy. The World Health Organization (WHO) recommendation to do so with eight weekly doses of 45 mg primaquine base does not come with evidence of safety among the most severe G6PD deficiency variants, which happen to be most common where *P. vivax* occurs in greatest abundance (south and southeast Asia).^12^ Finally, a recent study suggests that relatively common mutations to 2D6 cytochrome P-450 leaves patients partially or fully exposed to risk of relapse, despite primaquine therapy.^13^

An accompanying editorial by Price^14^ regarding a clinical trial of primaquine therapy for vivax malaria in Peru addresses the even broader problem of the effectiveness of primaquine therapy in the real world, where G6PD screening is rarely available and compliance to a 14-day dosing regimen is quite unlikely. The issues of mortality risk, hypnozoites, G6PD deficiency, and primaquine therapy all enmesh within a complex technical weave that may, at first, be difficult to grasp and understand. Success at doing so, however, illuminates an important raw fact—we have nearly no ability to mitigate the burden of morbidity and mortality imposed by the hypnozoite reservoir of *P. vivax*. The work of Quispe and others^1^ serves to further show that this state of affairs carries very serious consequences.
